# The yeast Gdt1 protein mediates the exchange of H^+^ for Ca^2+^ and Mn^2+^ influencing the Golgi pH

**DOI:** 10.1016/j.jbc.2023.104628

**Published:** 2023-03-22

**Authors:** Antoine Deschamps, Louise Thines, Anne-Sophie Colinet, Jiri Stribny, Pierre Morsomme

**Affiliations:** UCLouvain, Louvain Institute of Biomolecular Science and Technology (LIBST), Group of Molecular Physiology, Louvain-la-Neuve, Belgium

**Keywords:** GDT1, Golgi, proton transport, exchanger, organellar pH homeostasis, Saccharomyces cerevisiae, UPF0016

## Abstract

The *GDT1* family is broadly spread and highly conserved among living organisms. *GDT1* members have functions in key processes like glycosylation in humans and yeasts and photosynthesis in plants. These functions are mediated by their ability to transport ions. While transport of Ca^2+^ or Mn^2+^ is well established for several *GDT1* members, their transport mechanism is poorly understood. Here, we demonstrate that H^+^ ions are transported in exchange for Ca^2+^ and Mn^2+^ cations by the Golgi-localized yeast Gdt1 protein. We performed direct transport measurement across a biological membrane by expressing Gdt1p in *Lactococcus lactis* bacterial cells and by recording either the extracellular pH or the intracellular pH during the application of Ca^2+^, Mn^2+^ or H^+^ gradients. Besides, *in vivo* cytosolic and Golgi pH measurements were performed in *Saccharomyces cerevisiae* with genetically encoded pH probes targeted to those subcellular compartments. These data point out that the flow of H^+^ ions carried by Gdt1p could be reversed according to the physiological conditions. Together, our experiments unravel the influence of the relative concentration gradients for Gdt1p-mediated H^+^ transport and pave the way to decipher the regulatory mechanisms driving the activity of *GDT1* orthologs in various biological contexts.

Over the last years, a new family of membrane transporters has emerged in the literature, namely, the Uncharacterized protein family 0016 (UPF0016), also referred to as the *GDT1* family in Uniprot and InterPro databases (InterPro accession number: IPR001727). This protein family has been highly conserved during evolution and members of the family are found in all kingdoms of life, from prokaryotes to humans, through archea, plants, or yeasts (1). The human ortholog of the family—TMEM165—when mutated is responsible for a subtype of congenital disorder of glycosylation (2). All members of this protein family are characterized by the presence of one or two copies of a signature E-x-G-D-(K/R)-(T/S) motif located in predicted transmembrane spans of these proteins. When containing only one copy of this motif, proteins are thought to dimerize to form functional complexes. When having two copies, the topological organization of the protein displays an antiparallel topology, with two repeats of three transmembrane spans oppositely oriented in the membrane (1). This topology is typical for secondary transporters, such as the cation/Ca^2+^ exchangers (3).

Most studies on the yeast Gdt1p and its orthologs have pointed out to a Ca^2+^ and/or Mn^2+^ transport activity or implications in the homeostasis of those cations (4). Several articles documented that the transport of Ca^2+^ and Mn^2+^ could occur against their concentration gradient (5, 6, 7, 8). This implies that a driving force would be required to transport those cations across the biological membrane where they reside. A few studies further suggested that the H^+^ gradient could serve as driving force for Ca^2+^/Mn^2+^ transport. Among others, this hypothesis arises from the observation that the lysosomal pH of human patients with mutated TMEM165 is disturbed (9). Recently, it was also shown that the Golgi pH is decreased in cells depleted for TMEM165 (10). In yeast, phenotypical measurements of strains combining *GDT1* and vacuolar-type ATPase (V-ATPase) subunit *STV1* deletions reinforced this hypothesis because *GDT1* deletion partially counteracts *stv1Δ* sensitivity to the Ca^2+^ chelator 1,2-bis(*o*-amino phenoxy)ethane-*N,N,N′,N′*-tetra acetic acid (BAPTA) (11). Gdt1p was also shown to be involved in Ca^2+^ dissipation after transient elevation in the cytosolic Ca^2+^ concentration and that this dissipation requires a proper H^+^ gradient across the Golgi membrane (12). A role in proton homeostasis was also highlighted for plant orthologs; deletion of the chloroplastic *ccha1/pam71* in *Arabidopsis thaliana* affects its cytosolic pH, increases sensitivity to external pH variations, and alters pH gradient across the thylakoid membrane (7, 13). However, in all aforementioned studies, the proton transport activity has never been directly recorded, and one cannot exclude that those pH perturbations could derive from other dysregulations. Indeed, Ca^2+^ cations are well-known secondary messengers and Mn^2+^ cations serve as cofactor for several enzymes, in addition to play a role in the redox regulation (14, 15, 16), and these could indirectly influence the neighboring pH.

Here, we developed two different methods to unambiguously assess whether the yeast Gdt1p ortholog transports H^+^. At first, direct transport measurements were set up using a heterologous expression system, that is, *Lactococcus lactis* bacteria, as a host for Gdt1p expression and transport measurements. To highlight H^+^ transport activity in this system, both internal and external pH were recorded using intracellular superfolder-pHluorin (sfpHluorin), a modified GFP sensitive to pH (17, 18), or extracellular pH-meter. Second, *in vivo* pH measurements were performed directly in yeast, both in the cytosol and in the Golgi lumen, using cytosolic expression of the pHluorin and a Golgi-localized pH sensor derived from the same pHluorin that was recently developed in-house (19).

Those experiments bring the first direct evidence that H^+^ is transported by a *GDT1* family member across biological membranes and confirm the exchange of H^+^ ions for Ca^2+^ and Mn^2+^ divalent cations. Finally, it brings new information about the physiological role of the yeast Gd1p ortholog, which together with the V-ATPase plays a role for the control of the pH within the Golgi.

## Results

### Gdt1p mediates H^+^ transport across biological membranes

**Figure 1:**
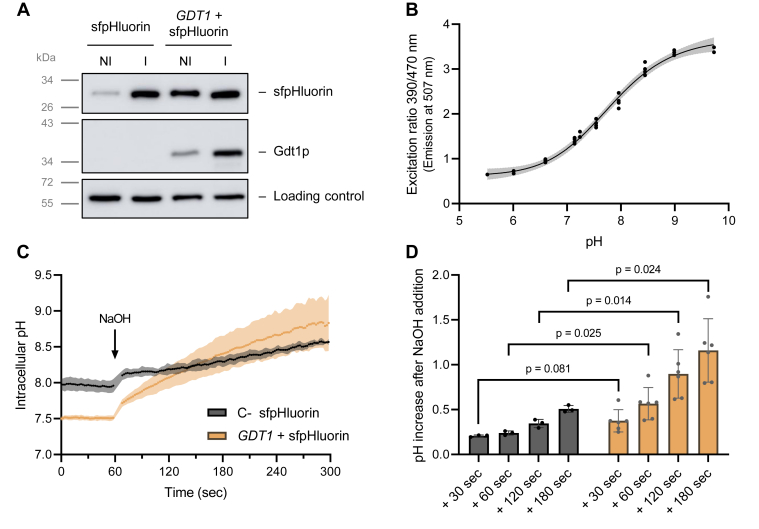
**Gdt1p transports protons through biological membranes.***A*, *Lactococcus lactis* cells are used as a tool to express Gdt1p at the membrane together with the sfpHluorin pH-sensor in the cytosol of the cell. Protein expression is induced by the addition of nisin for 2 h in the culture medium. A comparison of non-induced (NI) and induced (I) cells expressing only sfpHluorin or both proteins (GDT1 + sfpHluorin) was done by *Western blotting* on protein extracts. The sfpHluorin protein is detected by α-GFP antibodies, Gdt1p by α-Gdt1p antibodies, and the loading control consists of an undetermined endogenous protein detected by α-Gdt1p antibodies. *B*, Calibration curve of the sfpHluorin expressed in *L. lactis*. Cells collected 2 h after induction were washed, permeabilized by digitonin, and incubated in different pH buffers. After blank subtraction (cells devoid of sfpHluorin), the 390/470 nm excitation ratio (with emission recorded at 507 nm) is plotted against pH. A four-parameter sigmoidal curve is deduced from experimental data. *N* = 4, standard curve is represented with a 99% confidence interval. *C*, intracellular pH measurements over time of *GDT1* expressing cells (*GDT1* + sfpHluorin) and the corresponding negative control (C- sfpHluorin) after extracellular alkalinization by the addition of 10 mM sodium hydroxide (NaOH). This generates an H^+^ gradient with lower external [H^+^] compared to the intracellular [H^+^]. Then, the internal pH was monitored for 4 additional minutes. *N* = 3 to 6. *D*, quantification of pH increase at different time points (+30 s, + 60 s, + 120 s, + 180 s) compared to the average pH recorded during the first 60 s of the measurement, prior to sodium hydroxide addition. *N* = 3 to 6. Two-way ANOVA with Geisser-Greenhouse correction and multiple comparison *via* a Bonferroni test. sfpHluorin, superfolder-pHluorin.

A heterologous expression system using *L. lactis* as bacterial host was previously used in our laboratory to measure Ca^2+^ and Mn^2+^ influx mediated by Gdt1p (5, 6). We took advantage of the same heterologous expression system to assess H^+^ transport, by concomitantly expressing Gdt1p and an intracellular pH sensor, the genetically encoded sfpHluorin (18). Both Gdt1p and sfpHluorin proteins were expressed *via* a nisin-inducible expression system. Their expression level was checked by *Western blotting*, confirming that both were correctly expressed after nisin induction (Fig. 1*A*). It is noteworthy that, in non-induced condition, sfpHluorin production differs when co-expressed with Gdt1p or when expressed alone. After induction, sfpHluorin expression is similar in both strains. Anyway, differences in amount of sfpHluorin do not impair pH determination as measurements are handled in a ratiometric manner. *Per contra*, blank measurements that consist in cells that do not express sfpHluorin were systematically subtracted in order to reveal the specific fluorescent excitation spectrum of sfpHluorin (Fig. S1*A*). Those background subtracted data were used for further pH calculation. An *in vivo* calibration of the system was performed by using different pH buffers and H^+^/K^+^ nigericin ionophore in order to equilibrate internal with external pH (Figs. 1*B* and S1*B*). This effective tool was then used for *in vivo* intracellular pH measurements.

**Figure 2:**
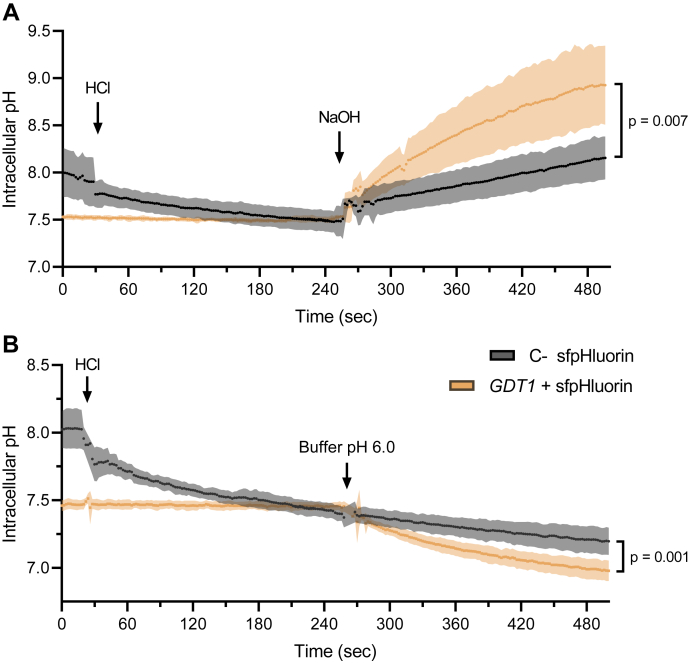
**Reversible transport of protons is mediated by Gdt1p in bacterial cells.** In order to correct for differences in initial internal pH between the strains producing or not producing Gdt1p, 10 mM of HCl was first added to the negative control (C- sfpHluorin). Four minutes later, alkalinization (*A*) or acidification (*B*) of the extracellular medium was performed. *A*, NaOH was added to both cell suspensions: 10 mM was added to *GDT1* + sfpHluorin expressing cells and 20 mM to the negative control, in order to neutralize the NaOH and to create the same pH balance for both strains. *B*, The extracellular pH was set at pH 6.0 by the addition of MES buffer at a final concentration of 50 mM. *N* = 5 to 7. For (*A* and *B*), the final pH values were statistically compared with an unpaired *t* test. sfpHluorin, superfolder-pHluorin.

The intracellular pH was measured over time after the initiation of pH gradients, either by the addition of NaOH, or by the addition of HCl or an acidic buffer, in the extracellular medium. The initial *L. lactis* intracellular pH recorded in our experiments (Figs. 1*C* and 2, *A* and *B*) is similar to pH values reported previously in the literature, which were in between 7.3 and 8.2 for various lactic acid bacteria and about 7.8 for *L. lactis* (20, 21). Strikingly, at the zero timepoint, the intracellular pH is lower in Gdt1p-expressing cells than in control cells producing only the sfpHluorin sensor. This may reflect Gdt1p-mediated H^+^ transport activity prior to pH recordings.

After recording the basal pH at 60 s, 10 mM of NaOH was added to the extracellular medium and the intracellular pH was monitored for 4 additional minutes (Fig. 1*C*). This addition creates a *in to out* [H^+^] gradient and protons are inclined to exit cells to equilibrate their concentration on either side of the membrane. Remarkably, the intracellular pH of cells expressing Gdt1p is increased in a higher magnitude and at a faster rate than control cells, indicating a larger H^+^ efflux (Fig. 1, *C* and *D*).

As the initial pH was not similar in *GDT1*-expressing cells and control cells, we also conducted measurements in which control cells were pre-treated by adding 10 mM HCl in the extracellular medium, in order to start measurements from the same intracellular pH. After a 4-min incubation, the internal pH of the control cells reaches roughly the same value as that of *GDT1*-expressing cells. Then, the subsequent addition of NaOH (+10 mM NaOH to *GDT1*-expressing cells; + 20 mM NaOH to control cells, to neutralize the 10 mM HCl and create a 10 mM excess of NaOH in the extracellular medium) leads again to a faster and more pronounced intracellular alkalization in *GDT1*-expressing cells than in control cells (Fig. 2*A*).

In order to monitor putative protons influx, we imposed an opposite gradient by the addition of HCl to *L. lactis* cell suspension and similarly recorded internal pH over time (Fig. S2*A*). Even if modest, the quantification of the pH decrease reveals a *GDT1*-specific effect, the cytosol acidifying a bit more in *GDT1*-expressing cells than in control cells (Fig. S2*B*). Again, similar to the previous experiment, the same initial intracellular pH was attained in both strains by the addition of extracellular HCl to control cells. Then, the subsequent application of an *out to in* [H^+^] gradient identical for both strains, by adding a concentrated pH buffer at value 6.0, inclines protons to further enter cells. This time, measurements highlight that *GDT1*-expressing cells import protons to a larger extent than control cells, as reflected by their lower final intracellular pH (Fig. 2*B*). Together, those experiments are the first direct evidence that Gdt1p is able to transport protons through biological membranes.

### The pH gradient influences Ca^2+^ and Mn^2+^ Gdt1p transport activity

As protons are thought to be the counterions of Ca^2+^ and Mn^2+^ during Gdt1p-mediated transport activity, a variation in the pH gradient across the Gdt1p-containing phospholipidic membrane should influence the transport of those two cations, as observed for other H^+^/Ca^2+^ antiporters (22, 23). To assess this assumption, we measured Ca^2+^ and Mn^2+^ import in *L. lactis* cells containing the fluorescent probe Fura-2, as previously performed in our laboratory (5, 6), at different extracellular pH.

**Figure 3:**
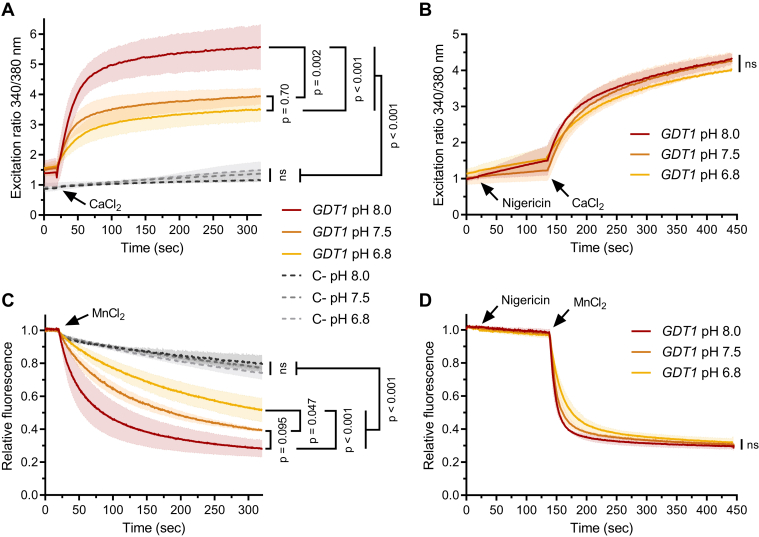
**Ca**^**2+**^**and Mn**^**2+**^**Gdt1p-mediated influx is affected by the pH gradient.***Lactococcus lactis* cells were grown, nisin-induced, and collected as in Figure 1. Then, cells were treated to internalize Fura-2. Finally, they were washed twice with a washing buffer (100 mM KCl, 1 mM MgCl_2_, 1 mM EGTA, 50 mM Tris at pH 6.8, 7.5 and 8.0) and resuspended in the corresponding buffer devoid of EGTA but previously passed through a calcium sponge resin to minimize the free Ca^2+^ concentration. *A* and *C*, Time-course Ca^2+^ and Mn^2+^ measurements of cells producing Gdt1p (*GDT1*) were performed at different extracellular pH, respectively, pH 6.8, 7.5, and 8.0. Ca^2+^ influx is provoked by the addition of 25 μM CaCl_2_ in the extracellular medium and is deduced from the calculation of the 340/380 nm excitation ratio, while 10 μM MnCl_2_ is added to induce Mn^2+^ influx, which is correlated to quenching during excitation at 360 nm. The Fura-2 fluorescence emission is invariably recorded at 510 nm. A negative control (C-) is also performed with cells that do not express Gdt1p. *N* = 3. The final data of each series were analyzed by one-way ANOVA and multiple comparisons Tukey test. *B* and *D*, To neutralize the pH gradient, the H^+^/K^+^ nigericin ionophore is added to the cell suspension at a concentration of 0.5 μg/ml 2 min before the addition of Ca^2+^ or Mn^2+^, and the cation influx measurement is performed as described previously. *N* = 3. The final data of each series were analyzed by one-way ANOVA and multiple comparisons Tukey test. All *p* values from the pairwise comparison are shown in Table S1.

Shortly, *L. lactis* cells were incubated with the esterified Fura-2-AM, which is able to cross membranes. Once inside the cell, intracellular esterases hydrolyze the ester function of the molecule, resulting in a charged form of Fura-2 that is consequently trapped in the cell. Internal Ca^2+^ concentration is then measured in a ratiometric manner, with two excitation peaks at 340 and 380 nm which are inversely affected by the Ca^2+^ concentration. An increase in the 340/380 excitation ratio indicates an increase in the intracellular Ca^2+^ concentration. Mn^2+^ import into the cells can also be detected using Fura-2 because its fluorescence is quenched by this cation. Ca^2+^ and Mn^2+^ transport measurements were carried out by the addition of CaCl_2_ or MnCl_2_ in the extracellular medium of cells incubated in buffers at pH 6.8, 7.5, and 8.0. The higher the extracellular pH, the higher the 340/380 ratio increases after CaCl_2_ addition, reflecting a larger increase of the intracellular Ca^2+^ concentration (Fig. 3*A*). For Mn^2+^, the higher the extracellular pH, the more pronounced the quenching, reflecting similarly a larger uptake of Mn^2+^ (Fig. 3*C*). Together, this indicates that the proton gradient, when more favorable to H^+^ efflux, allows a quicker Ca^2+^ and Mn^2+^ influx. This is in line with the hypothesized Ca^2+^-Mn^2+^/H^+^ exchange activity of Gdt1p.

As a control, cells non-expressing Gdt1p were used for analogous measurements. The basal transport activity recorded with those cells is not modified by changes in the extracellular pH, as the 340/380 excitation ratio or the quenching of the fluorescence is similar whatever the extracellular pH (Fig. 3, *A* and *C*). As another control, the addition of the ionophore nigericin, which dissipates the H^+^ gradient across membranes, results in a similar transport activity of Gdt1p for Ca^2+^ or Mn^2+^ whatever the external pH (Fig. 3, *B* and *D*). This confirms that the proton gradient itself modulates Ca^2+^ and Mn^2+^ Gdt1p transport activity.

### Gdt1p operates as a Ca^2+^-Mn^2+^/H^+^ exchanger

**Figure 4:**
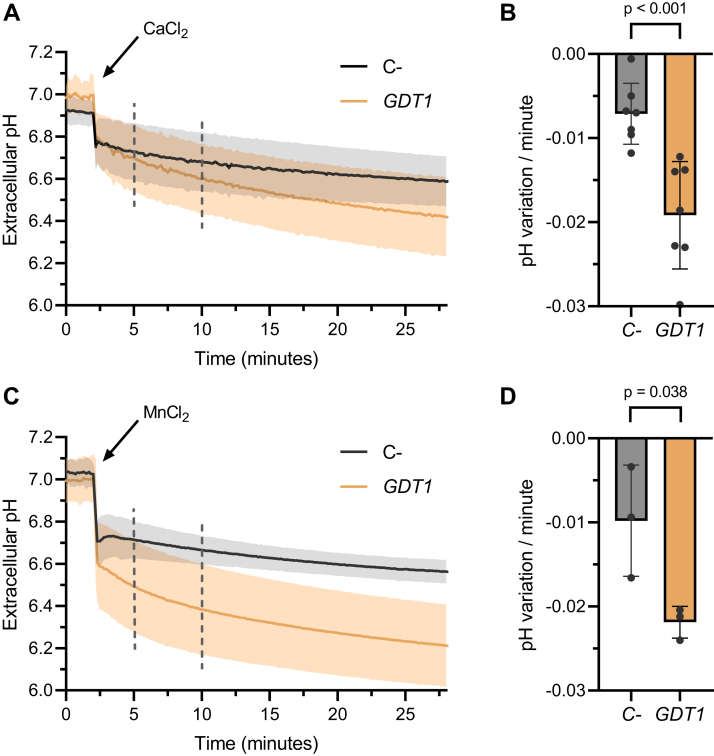
**Exchange of H**^**+**^**against Ca**^**2+**^**and Mn**^**2+**^**divalent cations mediated by Gdt1p.***A* and *C*, The extracellular pH of *Lactococcus lactis* cells expressing Gdt1p (*GDT1*) or transformed with an empty plasmid (C-) is compared during the addition of Ca^2+^ or Mn^2+^ in the extracellular medium. Cells collected by centrifugation are washed with a low concentrated pH buffer (1 mM Tris pH 7.0). Then, the extracellular pH is recorded over time *via* a pH meter. After recording the baseline for 2 min, 20 mM CaCl_2_ (*A*) or 20 mM MnCl_2_ (*C*) is added in the extracellular medium and the measurement is continued for up to 30 min. The dotted lines represent the period of time considered for slope measurement. *N* = 3 or 4. *B* and *D*, Quantification of the slope of pH decrease (pH variation per minute) after CaCl_2_ or MnCl_2_ addition in the extracellular medium, measured between 5 to 10 min of the time-course measurement (between *dotted lines* in (*A* and *C*)). *N* = 3 to 7. Unpaired *t* test were used as statistical analyses.

In order to directly measure Ca^2+^ or Mn^2+^ exchange with H^+^, we measured the extracellular pH of *L. lactis* cells expressing Gdt1p during Ca^2+^ and Mn^2+^ influx with a classical pH-meter. In case of exchange activity, the entry of Ca^2+^ or Mn^2+^ should trigger an efflux of H^+^ and therefore induce an acidification of the external medium. Because the internal pH is presumably highly buffered by a large number of weak acids and bases (24) and by several control mechanisms involving the activation of various H^+^ pumps as a result of intracellular pH sensing (25), the occurrence of internal pH changes requires strong driving forces, for example, as performed in Figures 1*C* and 2*A*. Here, by resuspending the cells into a weakly buffered solution, it facilitates the detection of external pH variations. Adding 20 mM CaCl_2_ or 20 mM MnCl_2_ in the extracellular medium first causes a pH drop in both the negative control and the Gdt1p-expressing strains. After this initial pH drop, the external pH continues to decrease in a different manner for the two strains, the external pH decreasing faster in cells expressing Gdt1p than in control cells (Fig. 4, *A*–*D*). This likely reflects the Ca^2+^-Mn^2+^/H^+^ exchange activity mediated by Gdt1p.

### Steady state Golgi and cytosolic pH are not substantially regulated by Gdt1p in *Saccharomyces cerevisiae*

**Figure 5:**
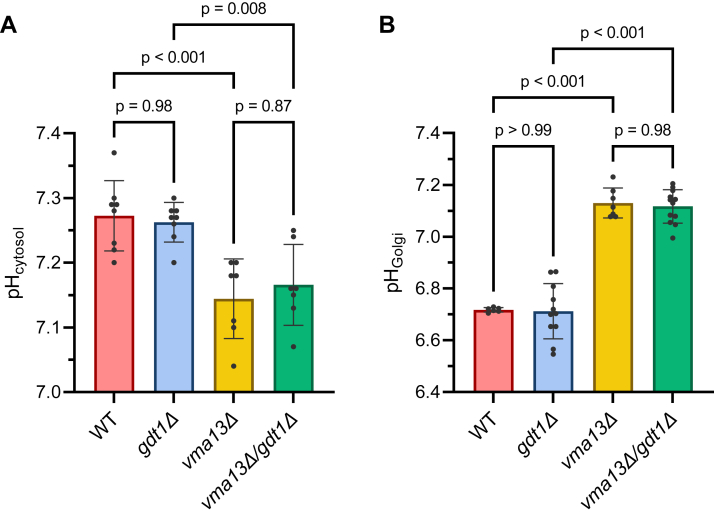
**The deletion of *GDT1* does not disturb the intracellular pH values of exponentially growing cells.** (*A*) Cytosolic pH and (*B*) Golgi pH measurements in steady-state growth conditions. *WT*, *gdt1Δ*, *vma13Δ*, and *vma13Δ/gdt1Δ* strains expressing cytosolic and Golgi pH probes were cultivated in synthetic media buffered at pH 5.0 (50 mM MES) and collected during mid-log growth phase for direct pH measurements. *N* = 7 or 8 for pH_cytosol_ and *N* = 6 to 12 for pH_Golgi_. One-way ANOVA and multiple comparisons Tukey tests were used as statistical analyses.

The previous measurements were performed in a heterologous expression system. Although it is remarkably suitable to measure direct transport activities, it does not unveil the physiological function of Gdt1p in its natural environment, in *Saccharomyces cerevisiae.* Because of its localization at the Golgi membrane, Gdt1p could potentially influence both cytosolic and Golgi pH. Therefore, we monitored the pH in those two subcellular compartments *via* the expression of the original pHluorin (17) in the cytosol and thanks to a pHluorin variant targeted to the Golgi lumen (19). When cells are cultivated in a synthetic medium and collected during exponential growth for direct pH measurement, the deletion of *GDT1* does not modify the cytosolic pH compared to the *WT* strain (Fig. 5*A*). It is not totally unexpected, as many other transporters are operating H^+^ import or export, from and to the cytosol (25), and because of the inherent buffering capacity of the cytosol (26). Besides, Golgi pH measurements disclose that *GDT1* deletion does not significantly affect the luminal Golgi pH either (Fig. 5*B*). This implies that, under physiological conditions, Gdt1p Ca^2+^-Mn^2+^/H^+^ exchange activity is not a predominant driver of Golgi luminal acidification. This role is probably mostly fulfilled by the V-ATPase alongside the secretory pathway acidification since the Golgi pH becomes almost neutral in the *vma13Δ* strain (Fig. 5*B*). Deleting this major H^+^ pump would reveal the Gdt1p role in cytosol and/or Golgi pH homeostasis. The concomitant deletion of *GDT1* and *VMA13* does not change pH values of the cytosol or the Golgi compared to *VMA13* deletion alone (Fig. 5, *A* and *B*). Altogether, those measurements indicate that the determination of the Golgi pH value during exponential growth is not the main physiological role of Gdt1p.

### During physiologic pH modifications, Gdt1p participates in the homeostasis of the Golgi pH

Most commonly, exchangers and symporters are described as having a high velocity, while pumps can produce larger concentration gradients but in a slower fashion (27). Therefore, the coexistence in the same biological membrane of transporters with common substrates but different transport mechanisms is complementary. For example, after the cytosolic Ca^2+^ pulse that mediates intracellular signaling, two yeast vacuolar Ca^2+^-transporters, the ATPase Pmc1p and the exchanger Vcx1p, collaborate to restore the resting cytosolic Ca^2+^ concentration (28). Vcx1p can rapidly transport a large amount of substrate at first, followed by a slower recovery of the resting state displaying expanded concentration gradients, thanks to the activity of Pmc1p. In mammals, this complementary role of transporters with different mechanisms is illustrated by the plasma membrane–localized PMCA Ca^2+^-ATPase and the NCX Na^+^/Ca^2+^ exchanger, which collaborate for the same purpose (29). Furthermore, secondary transporters and ATPases have different driving forces, and they can perform transport in different physiological statuses which is advantageous for cells to maintain homeostasis.

**Figure 6:**
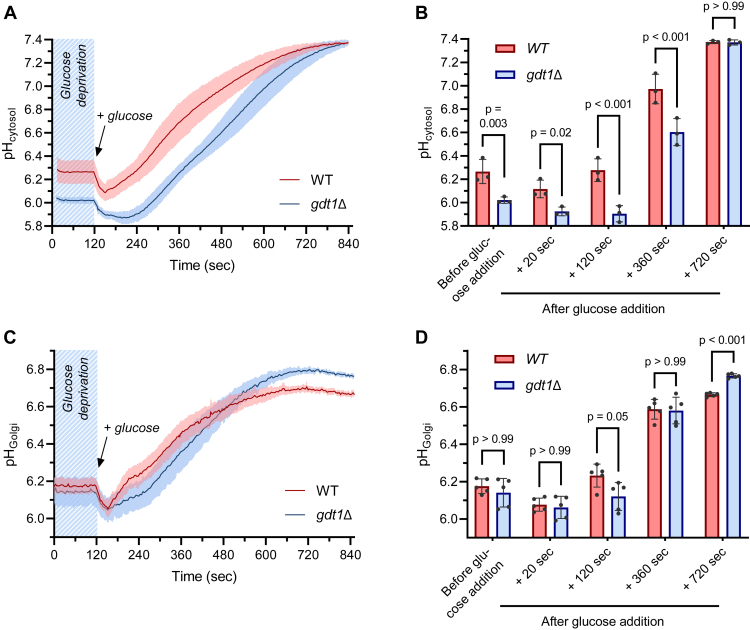
**Gdt1p influences the Golgi pH during glucose deprivation/re-addition.** (*A*) Cytosolic pH and (*C*) Golgi pH measurements during glucose deprivation/re-addition time-course assay. Cells were grown to mid-log phase in synthetic medium buffered at pH 5.0 (50 mM MES), collected, and washed 3 times in glucose deprived medium. After 60 min incubation at 28°C in glucose deprivation condition, cells were used for fluorescence measurement. The baseline was recorded during 2 min, then glucose was added to a final concentration of 2% and the Golgi or cytosolic pH were measured for 12 additional minutes. (*B*) Quantification of the cytosolic pH and (*D*) quantification of the Golgi pH at different time points before and after (+20 to + 720 s) glucose addition, from the time-course experiments presented in (*A* and *C*). *N* = 3 for pH_cytosol_, *N* = 5 for pH_Golgi_. Two-way ANOVA statistical analysis with Bonferroni multiple comparisons test.

Here, we hypothesize that Gdt1p could be important for cells to rapidly react during intracellular pH changes in complement to the predictably slower V-ATPase. A well-known procedure that triggers cytosolic pH fluctuations is the glucose deprivation—glucose addition process (30, 31). Cells deprived of carbon sources slowly acidify the cytosol for 30 to 60 min. If glucose is added back to the growth media, it is rapidly imported into the cell where glycolysis and metabolic activity resta rt. This provokes a transient additional acidification of the cytosol, possibly due to the production of H^+^ by glycolysis. Conjointly, the availability of glucose is detected by a set of dedicated sensors and signaling mechanisms that eventually lead to the activation of H^+^-ATPases by increasing the affinity of the plasma membrane Pma1p for ATP (32, 33) and by inducing the assembly of the V-ATPase in internal membranes (30, 34). Those pumps will extrude protons outside the cytosol, leading to an increase in its pH value until a new steady state level is reached. When performing such a process in the *gdt1Δ* strain and continuously recording the cytosolic pH using the pHluorin, one could observe that the trend is similar to the *WT* strain (Fig. 6*A*). After the deprivation period, the cytosolic pH is slightly lower in the *gdt1Δ* strain. Once glucose is added back, one can also observe a later recovery of the steady state cytosolic pH in the *gdt1Δ* strain compared to the *WT* (Fig. 6*B*), which may be directly due to the H^+^ transport activity of Gdt1p or may result from indirect effects of Gdt1p on the Ca^2+^ signaling process. In the literature, it is well described that a transient elevation in the cytosolic Ca^2+^ concentration occurs following glucose re-addition to starved cells and that the activation of H^+^-ATPases, pH fluctuations, and Ca^2+^ signaling processes are tightly intertwined (12, 35, 36). It has also been shown that *GDT1* deletion slightly affects the intensity of this transient elevation in the cytosolic Ca^2+^ (37). Hence, *GDT1* deletion, by affecting the Ca^2+^ signalling process, may provoke a delay in the recovery of the steady state cytosolic pH. Such a delay of pH recovery in the *gdt1Δ* strain also comes out when measuring the Golgi pH during a similar process (Fig. 6*C*). It is especially noticeable when considering the time point 120 s after glucose addition (Fig. 6, *B* and *D*). Finally, by the end of the recovery, the steady state cytosolic pH values recorded in *gdt1Δ* and *WT* strains are not statistically different.

By recording the Golgi pH during an identical glucose deprivation, re-addition procedure, we observe curves with a similar shape. Basically, fluctuations in the Golgi pH are mirroring the cytosolic pH fluctuations. Nevertheless, the pH increase is much more pronounced in the cytosol, with close to 1 pH unit increase, while there is only a 0.5 pH unit increase in the Golgi lumen of the *WT* strain. When comparing the Golgi pH fluctuations of *WT* and *gdt1Δ* strains, we observe that the Golgi pH reaches a higher value in the *gdt1Δ* strain than in the *WT* strain by the end of the recovery (Fig. 6, *C* and *D*). This observation strongly suggests that Gdt1p participates in the transport of protons toward the Golgi lumen during those pH fluctuations.

## Discussion

Several studies documented the role of *GDT1* family members in pH homeostasis but without demonstrating direct H^+^ transport. In this study, using *L. lactis* as a heterologous expression system, proton transport activity mediated by the yeast Gdt1p ortholog was investigated. At first, we should note that *L. lactis* cells expressing Gdt1p display a lower intracellular pH in basal conditions, before applying H^+^ gradient than control cells that do not express Gdt1p (Figs. 1*C*, 2, *A* and *B* and S2*A*). A possible explanation for this is the composition of the washing buffer that, by containing EGTA (1 mM EGTA, 100 mM KCl, 1 mM MgCl_2_, 20 mM MOPS pH 7.4), likely generates a *in to out* [Ca^2+^] gradient. Hence, if Gdt1p exchanges Ca^2+^ for H^+^ ions, Ca^2+^ efflux along this gradient will trigger the acidification of *L. lactis* cytosol prior to pH recordings. Furthermore, as the washing buffer is set up at pH 7.4, H^+^ transport activity mediated by Gdt1p will result in an internal pH value close to the one of the extracellular buffer, as observed in the recordings of the initial pH baselines.

Then, pH monitoring during the application of proton gradients across cytoplasmic membrane revealed larger intracellular pH changes in *GDT1*-expressing cells than in control cells (Fig. 1, *C* and *D* and S2, *A* and *B*), demonstrating Gdt1p-related H^+^ transport activity. Interestingly, such Gdt1p-specific effect are noticed both when *in to out* or *out to in* pH gradients were applied. At least two different reasons could explain it. If we consider unidirectional transport mediated by each individual protein, one explanation is that part of the protein is oriented with one topology and part with the opposite topology within the *L. lactis* membrane. However, membrane proteins usually display a preferential topology dictated by the positive inside rule, both in prokaryotes and in eukaryotes (38). Gdt1p displays enrichment in positively charged lysines and arginines on one side of its predicted transmembrane domain and was therefore predicted to adopt one specific topology in the yeast Golgi membrane, which was validated in our lab by a protease degradation assay (1). Therefore, we similarly anticipate having a preferred Gdt1p topology within the *L. lactis* membrane. Thus, the second explanation is that Gdt1p could transport protons either in one or the other direction, according to the pH gradient that is applied across the membrane. Secondary transporters, like all enzymes, are catalyzers of biochemical reactions. Their role is to fasten the establishment of thermodynamic equilibrium, but they do not dictate the direction of it and they can theoretically operate in both ways. Of course, in biological systems, the direction of operability is driven by the concomitant concentration of substrates and products or by the concentration of transported molecules at both sides of a biological membrane in the specific case of membrane channels and transporters. Here, by using a heterologous expression system and by applying H^+^ concentration gradients in both directions with sufficient amplitude, one observes presumably reversible transport activity of Gdt1p.

Obviously, the expression of an exogenous membrane protein in *L. lactis* cells could affect the integrity of its accommodating phospholipidic bilayer, reducing the stringency of such hydrophobic barrier, or could deregulate endogenous membrane transporters. This possibility is even more conceivable because control cells that do not express Gdt1p display inherent H^+^ transport activity, as the addition of NaOH or HCl in the extracellular medium causes some increase or decrease of the intracellular pH, respectively. Therefore, as we speculated that Gdt1p could exchange H^+^ ions for Ca^2+^ and/or Mn^2+^ cations, we performed measurements where the transport activity of one substrate was recorded when the concentration gradient of its putative counterion was modified. We observed on one side that the larger the *in to out* proton gradient, the faster the Ca^2+^ or Mn^2+^ influx (Fig. 3, *A* and *C*), as already reported previously for Ca^2+^ (5). On the other side, the application of Ca^2+^ or Mn^2+^ *out to in* gradients induces protons efflux (Fig. 4, *A*–*D*). Henceforth, when the transport of one substrate is favored in one direction, it increases the transport of its presumed counterion in the opposite direction. Taken together, these data decisively demonstrate that Gdt1p conveys H^+^ ions and that this transport of H^+^ happens in exchange for Ca^2+^ and Mn^2+^ cations.

In order to clarify the physiological role of Gdt1p in its normal environment, that is, the Golgi membrane of *S. cerevisiae*, it is crucial to measure its activity in its native environment. Therefore, we measured the pH of the cytosol and the Golgi lumen, thanks to suitable pHluorin variants in various conditions. Curiously, *GDT1* deletion does not significantly impair physiological steady state pH values, neither in the cytosol nor in the Golgi. As there are other important pH regulators, such as the V-ATPase, which is the main protein acting to acidify the secretory pathway (39, 40), Gdt1p is probably dispensable for Golgi acidification in favorable growth conditions. Even in V-ATPase deficient cells where the cytosolic pH is slightly lower than in *WT* cells and where the Golgi pH is almost neutral, the role of Gdt1p for the maintenance of pH homeostasis does not pop up.

However, when a quick response and a high rate of H^+^ transport are required, the presence of a secondary transporter could be beneficial for the cell, since the transport velocity of secondary transporters is usually higher than that of ATP-driven transporters (27). In yeast, pH homeostasis is intricate to glucose availability. When recording cytosol and Golgi pH during a glucose deprivation—readdition experiment—it is striking that pH fluctuations in the Golgi mimics the fluctuations that occur in the cytosol. In biological systems, several weak acids and bases are membrane-permeant, such as ammonia, acetic, formic, or carbonic acid, and will therefore be involved in the establishment of cytosolic and organellar pH values. The permeability to solutes of the different phospholipidic membranes increases from the thinner ER membrane to the thicker plasma membrane (41, 42, 43). In complement to this inherited property, numerous membrane proteins could be involved in the transfer of protons across the membranes. At the early Golgi, our data suggest that some weak acids and bases could potentially cross the membrane quite easily, since Golgi pH fluctuations mimic so well cytosolic pH fluctuations. It is maybe complementary or part of a putative “proton-leak” channel that has not been identified to date (44). Still, some dedicated H^+^ transporters have to maintain a relative acidity within the Golgi lumen, especially once glucose is again available. While the V-ATPase is predominant for Golgi acidification, Gdt1p participates in such a process. Especially, when the cytosolic pH increases steeply after glucose re-addition, the increase in the Golgi pH is rapidly restrained. The deletion of *GDT1* mitigates this restriction since the final Golgi pH value attained in the *gdt1Δ* strain is significantly higher than the one of the *WT*.

**Figure 7:**
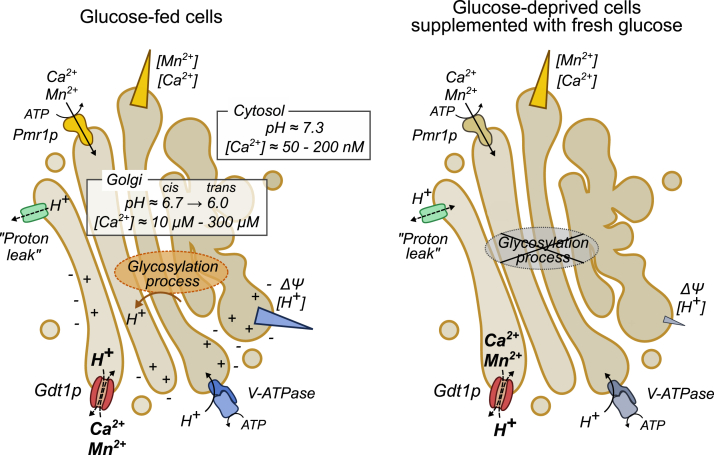
**Model of reversible transport activity mediated by Gdt1p.***Left part*: In favorable growth conditions, when cells are fed with glucose and ATP is readily available, both the V-ATPase and the Pmr1p are actively pumping protons and divalent cations, respectively, from the cytosol to the Golgi lumen. This generates a net gradient of Ca^2+^, Mn^2+^, and H^+^ as well as a positive inside membrane potential Ψ (39, 45). Moreover, the activity of glycosylation enzymes generates a release of protons within the Golgi lumen, hence participating in its acidification (11). With the hypothesis that several H^+^ ions are exchanged for a single Ca^2+^ or Mn^2+^ cation, Gdt1p would import Ca^2+^ and Mn^2+^ cations and export H^+^ in an energetically favorable manner. In those conditions, Gdt1p supports Pmr1p in the loading of the Golgi lumen with Ca^2+^ and Mn^2+^. Furthermore, the presumed H^+^ leak system, which participates in the efflux of H^+^ out of the Golgi (44), could be assisted by Gdt1p for this purpose. Right part: During glucose deprivation, yeast cells get their cytosol acidified and both the plasma membrane Pma1p H^+^-ATPase and the secretory pathway/vacuolar V-ATPase are maintained in an inactive state. At the same time, the glycosylation process is shut down. As passive equilibration mechanisms are in place, the Golgi to cytosol pH gradient and the membrane potential will decline until they get neutralized. When glucose is added back, the cytosolic pH increases rapidly (Fig. 6*A*). As a result, Gdt1p could revert its transport activity to take advantage of the Ca^2+^ and Mn^2+^ gradients, which are reminiscent from an earlier period and/or regenerated by Pmr1p, to import H^+^ ions from the cytosol to the Golgi lumen. Gdt1p would therefore participate in the preservation of some acidity within the Golgi lumen.

Therefore, we propose a model where Gdt1p usually transports Ca^2+^ and Mn^2+^ cations to the Golgi lumen by using the H^+^ gradient, possibly combined with the positive inside membrane potential (45) generated by the V-ATPase (Fig. 7, left pannel). In this situation, the role of Gdt1p is complementary to that of the Golgi-localized Ca^2+^/Mn^2+^-ATPase Pmr1p, which is to extrude any excess of cytosolic Ca^2+^ and Mn^2+^ and feed the secretory pathway with those cations (5, 6, 46, 47). Gdt1p has affinities for Ca^2+^ and Mn^2+^ cations (K_M_ = 15 μM for Ca^2+^, 83 μM for Mn^2+^ (6)) that are lower than the ones of Pmr1p (K_M_ = 0.1 μM for Ca^2+^ (48), 0.02 μM for Mn^2+^ (49)) but is probably characterized by transport kinetic and mechanism that may be beneficial for the cell in some conditions, as we will discuss it hereunder. The calcium being a cytosolic secondary messenger, it is essential that cells possess transporters able to rapidly cope with any increase in its cytosolic concentration to avoid messy signaling. Besides, there is increasing evidence that Ca^2+^ is involved in the control of vesicular trafficking (50, 51) by modulating SNAREs activity (52, 53, 54). Therefore, Gdt1p activity could be important for the proper regulation of Golgi vesicular trafficking by mediating rapid release or reuptake of Ca^2+^ into the Golgi lumen. Accordingly, *gdt1Δ* cells exposed to high extracellular Ca^2+^ display glycosylation defects that might reflect trafficking defects. As a corollary, in the *pmr1Δ* strain, *GDT1* deletion or overexpression is characterized by trafficking defects in a Ca^2+^-dependent manner (55). Hence, it is conceivable that local Ca^2+^ signaling events around Golgi compartments, which regulate intra-Golgi membrane trafficking (51), are disturbed when *GDT1* is deficient. For its part, manganese is a well-known regulator of redox status within biological systems (16) but also serves as a cofactor for several glycosylation enzymes in the Golgi apparatus (56, 57, 58). If it is not loaded sufficiently in the Golgi lumen, it is a cause of glycosylation disorder (59). In favorable growth conditions, Gdt1p may be a partner of Pmr1p to provide their Mn^2+^ cofactor to glycosylation enzymes.

However, Gdt1p-mediated Ca^2+^ and Mn^2+^ transport activity could affect Golgi homeostasis. Especially, the pH is a key parameter for most biochemical phenomena, as protonation or deprotonation of amino acids will directly influence the shape and therefore the functionality and the interactions of most proteins. The luminal acidification alongside the secretory pathway, from its “origin”, that is, the ER, to its “destination”, that is, exocytic vesicles and vacuoles, is important to maintain the organization of the secretory pathway (39, 60). Here, our data let us speculate that Gdt1p can use the transmembrane Ca^2+^ and Mn^2+^ gradients to convey protons from the cytosol to the Golgi lumen, in support of the V-ATPase, when cells are facing a rapid cytosolic pH increase (Fig. 6, *A* and *C*). Thanks to Gdt1p transport activity, the Golgi lumen does not experience a too intense pH increase and rather keeps some acidity in the Golgi lumen. Hence, we complete our model with a putative reverse transport activity (Fig. 7, right panel). Of course, we have to be cautious in the interpretation of the measurements, as we do not know how all the transporters act during such pH fluctuations, and as we cannot monitor all the ionic gradients simultaneously. Nevertheless, our hypothesis is that the reverse activity of Gdt1p, by participating in the maintenance of an acidic luminal Golgi pH, could help preserve the Golgi structure and ensures a fast renaissance of Golgi metabolic functions by avoiding losses of Golgi resident enzymes and mistargeting of incompletely modified cargoes, and could ensure a correct activity of mannosidases and glycosyltransferases. Accordingly, Gdt1p maybe provides some fitness advantage to its host cell.

Altogether, our results shed light on the mechanism of transport of the *S. cerevisiae* Gdt1p, which exchanges H^+^ ions for Ca^2+^ and Mn^2+^ cations. Our experiments also suggest that the transport activity of Gdt1p could be reversed according to the physiological conditions. Furthermore, it emphasizes the roles of Gdt1p for Golgi homeostasis and brings clues to a deeper understanding of how pH regulation within the Golgi lumen guides Golgi organization and ensures its functionalities, particularly regarding the glycosylation process. Eventually, solving Gdt1p transport mechanism could be transposed to other *GDT1* family members to better characterize their function in animals, bacteria, archaea, or plants.

## Experimental procedures

### Plasmids and strains

The plasmids used in this study are listed in Table 1. For expression of the sfpHluorin, a gift from Eckhard Boles (Addgene plasmid #115697) (18), in DML1 *L. lactis* cells (61), the gene was cloned between the NcoI and HindIII restriction sites of the pNZ8048 plasmid, with the addition of one extra glycine after the Start codon standing within the NcoI restriction site. Therefore, the gene is under the control of pNisA promoter. For concomitant expression of *GDT1* and sfpHluorin, both genes were inserted in the same plasmid in an operon-like manner with an intergenic sequence between the two coding sequences (intergenic sequence: AAAAATAAAGAGAGGAAAAAAAAC). Note that a NcoI restriction site initially present in the original *GDT1* sequence was removed by introducing a silent mutation. To express *GDT1* alone, it was cloned between the SphI and HindIII restriction sites. For yeast experiments, the pH-sensitive pHluorin was either expressed from the pZR4.1-pTEF1-pHluorin-tCYC1 plasmid for cytosolic expression or inserted in the genome of strains of interest for the Golgi-localized variants.

**Table 1:** Plasmids used in this study

Plasmid	Abbreviate name	Source
pNZ8048-pNisA-sfpHluorin	sfpHluorin	This study
pNZ8048-pNisA-GDT1-intergenic sequence-sfpHluorin	GDT1 + sfpHluorin	This study
pNZ8048-pNisA-GDT1	GDT1	Our laboratory
pNZ8048-pNisA-empty	C-	B. Poolman, Groningen, Netherlands
pRS315-pTPI-Mnn2(1–36)-HA-pHluorin(F64L;M153R)-tCYC1	Mnn2-HA-pHluorin∗∗	Our laboratory (19).
pRS315-pTPI-USER-tCYC1	Empty vector	Our laboratory
pZR4.1-pTEF1-pHluorin-tCYC1	pHluorin cytosol	R. Rao, Baltimore, MD, USA (62)

From the pRS315-pTPI-Mnn2(1–36)-HA-pHluorin(F64L;M153R)-tCYC1 plasmid, a DNA product of about 4.3 kb containing two coding sequences and their related promoter and terminator, pTPI-Mnn2(1–36)-HA-pHluorin(F64L;M153R)-tCYC1 and pLEU2-LEU2, was amplified by PCR and used for genomic insertion at the *his3Δ1* locus in relevant *S. cerevisiae* strains, as described in Deschamps *et al.* (19). As a blank for background fluorescence measurements, the *WT* strain was also transformed with the pLEU2-LEU2 selection marker only. All *S. cerevisiae* and *L. lactis* strains used in this study are listed in Table 2.

**Table 2:** Yeast and bacteria strains used in this study

Strain	Description	Source
*L. lactis* DML1	Evolved derivative of the NZ9000 strain (61)	B. Poolman, Groningen,Netherlands
*S. cerevisiae* BY4742 wild type	*Matα his3Δ1 leu2Δ0 lys2Δ0 ura3Δ0*	Euroscarf
*S. cerevisiae* BY4742 *gdt1Δ*	*Matα his3Δ1 leu2Δ0 lys2Δ0 ura3Δ0 gdt1::KanMX4*	Euroscarf
*S. cerevisiae* BY4742 *vma13Δ*	*Matα his3Δ1 leu2Δ0 lys2Δ0 ura3Δ0 vma13::KanMX4*	Euroscarf
*S. cerevisiae* BY *vma13Δ/gdt1Δ*	*Mata his3Δ1 leu2Δ0 lys2Δ0 ura3Δ0 vma13::KanMX4 gdt1::KanMX4*	Our laboratory
*S. cerevisiae* BY4742 wild-type + Mnn2-HA-pHluorin∗∗	*Matα leu2Δ0 lys2Δ0 ura3Δ0 his3Δ1::LEU2-pTPI-MNN2-HA-pHluorin∗∗-tCYC1*	This study
*S. cerevisiae* BY4742 wild-type + selection marker	*Matα leu2Δ0 lys2Δ0 ura3Δ0 his3Δ1::LEU2*	This study
*S. cerevisiae* BY4742 *gdt1Δ* + Mnn2-HA-pHluorin∗∗	*Matα leu2Δ0 lys2Δ0 ura3Δ0 his3Δ1::LEU2-pTPI-MNN2-HA-pHluorin∗∗-tCYC1 gdt1::KanMX4*	This study
*S. cerevisiae* BY4742 *vma13Δ* + Mnn2-HA-pHluorin∗∗	*Matα leu2Δ0 lys2Δ0 ura3Δ0 his3Δ1::LEU2-pTPI-MNN2-HA-pHluorin∗∗-tCYC1 vma13::KanMX4*	This study
*S. cerevisiae* BY *vma13Δ/gdt1Δ* + Mnn2-HA-pHluorin∗∗	*Mata leu2Δ0 lys2Δ0 ura3Δ0 his3Δ1::LEU2-pTPI-MNN2-HA-pHluorin∗∗-tCYC1 vma13::KanMX4 gdt1::KanMX4*	This study

### Cultivation of *L. lactis* and induction of *GDT1* and sfpHluorin

*Lactococcus lactis* cells were usually cultivated in anaerobic conditions, except if stated otherwise. They were grown at 28 °C in M17 broth according to Terzaghi (Merck Millipore) supplemented with 1 % glucose and with 10 μg/ml chloramphenicol for pNZ8048 plasmid maintenance. Expression of genes under the control of the pNisA promoter was induced by 2.5 μg/liter nisin addition during the log growth phase (optical density at 600 nm (OD_600_) = 0.4 to 0.5) and the culture was extended for two additional hours before harvesting cells. For anaerobic growth, fully filled closed tubes were used, with no agitation. When the sfpHluorin was produced, cell cultivation was performed in aerobic conditions during the induction period to allow correct folding and expression of the sfpHluorin. In that case, half-filled slightly open tubes were used for cultivation, with agitation.

### *In vivo* sfpHluorin calibration

For *in vivo* calibration of the sfpHluorin in *L. lactis*, 200 ml of cell suspension were harvested by 5 min centrifugation at 1500*g* at 28 °C. Cells were washed twice with 15 ml of washing buffer (100 mM KCl, 1 mM EGTA, 1 mM MgCl_2_, 20 mM MOPS pH 7.2) and resuspended in the same buffer in an appropriate volume to reach OD_600_ = 5.0. The cell suspension was distributed in 2 ml fractions in microtubes. After 1 min centrifugation at 5000*g*, and the cell pellets were resuspended in buffer solutions at different pH. Those buffers have the same composition as the washing buffer, except the buffering compound, which is MES (pH 5.5, 6.0, or 6.5), MOPS (pH 7.0 or 7.2), TRIS (pH 7.5, 8.0, 8.5, or 9.5), or *N*-cyclohexyl-3-aminopropanesulfonic acid (pH 9.0) and that nigericin K^+^/H^+^ ionophore was added at 0.5 μg/ml. Excitation spectra were measured from 340 to 490 nm, with emission recorded at 507 nm, with a JASCO FP8500 fluorimeter controlled by the Spectra Manager Software. After blank subtraction, *i.e.* cells that do not express the sfpHluorin probe, a four-parameters sigmoidal curve of the 390/470 nm excitation ratio as a function of the pH was determined.

### Intracellular pH measurements in *L. lactis*

For intracellular pH measurements, 50 ml of nisin-induced cells expressing the sfpHluorin were collected by 3-min centrifugation at 1500*g* at 28 °C, washed twice with 15 ml washing buffer (100 mM KCl, 1 mM EGTA, 1 mM MgCl_2_, 20 mM MOPS pH 7.4), and resuspended again in the same buffer to get OD_600_ = 10.0. Two milliliters of cell suspension were then used for fluorescence measurement with alternate excitations at 390 and 470 nm (emission at 507 nm) for 5 to 10 min. Cells were maintained at 28 °C and continuously stirred (300 rpm) during the measurement. After baseline recording for 60 s, different compounds were added to generate transmembrane pH gradients. Blank subtracted data, where a blank signal is the signal recorded with cells that do not express the sfpHluorin, were converted into intracellular pH values using the previously-established calibration curve.

### Extracellular pH measurements with *L. lactis*

Extracellular pH measurements were performed using a pH-meter. Briefly, *L. lactis* cells were grown for intracellular pH measurements, except that the strains used were expressing only *GDT1*, or no exogenous protein for the negative control. After 2 h of protein production induction, 100 OD_600_ units (where 1 OD_600_ unit approximately corresponds to 10^7^ cells) were collected by centrifugation and washed twice with 15 ml of an appropriate buffer (50 mM Tris pH 7.0). Then, a third washing was performed with a weakly buffered solution (1 mM Tris pH 7.0). The last resuspension was performed in 20 ml of the same weakly buffered solution and the cell suspension was transferred into a small beaker for extracellular pH measurement. During pH recording, continuous stirring was performed, and CaCl_2_, MnCl_2_, or MgCl_2_ (stock solution at 400 mM with 1 mM Tris pH 7.0) was added at the indicated time point. For data analysis, raw pH measurements as well as pH variation after cation addition were considered.

### Ca^2+^ and Mn^2+^ transport measurements in *L. lactis* at different extracellular pH

The *in vivo* Ca^2+^ and Mn^2+^ transport measurements were carried out using the fluorescent dye Fura-2/AM according to the method previously described by Thines *et al.* (6) with slight modifications. Briefly, *L. lactis* DML1 cells expressing *GDT1* or transformed with an empty plasmid were collected, washed three times, and incubated with 10 μM Fura-2/AM and 1.7 mM probenecid for 2 h at 28 °C with agitation. Then, cells were washed twice with the appropriate washing buffer solution (100 mM KCl, 1 mM MgCl_2_, 1 mM EGTA, 50 mM Tris-HCl pH 6.8, 7.5 or 8.0). The final pellet was resuspended in the corresponding buffer devoid of EGTA but previously passed through a calcium sponge resin (BAPTA chelator coupled to a polymer matrix; Invitrogen) in order to decrease as much as possible the free Ca^2+^ concentration. For Ca^2+^ transport measurements, two excitation wavelengths of 340 and 380 nm were used and the excitation ratio is plotted as an estimation of [Ca^2+^] (ratiometric measurement). For Mn^2+^ transport measurements, the excitation is performed at the isosbestic point of 360 nm and the quenching of fluorescence is used as a read-out for [Mn^2+^]. In both cases, fluorescence emission was recorded at 510 nm by a JASCO FP8500 fluorimeter controlled by the Spectra Manager software.

### Golgi and cytosol pH measurements in *S. cerevisiae* at steady state or during glucose pulse

For *in vivo* pH measurements in *S. cerevisiae*, cells expressing the Mnn2-HA-pHluorin∗∗ protein for Golgi pH measurements or the native pHluorin adapted to cytosolic pH measurements, as well as corresponding blank strains that do not express any fluorescent probe, were cultivated in Low Fluorescence synthetic defined medium (0.7 % yeast nitrogen base without amino acids, without folic acid and without riboflavin (ForMedium), 2 % glucose, supplemented with all amino acids except those used as selection marker, buffered with 50 mM MES pH 5.0, filter-sterilized). Cells were collected and measured as in Deschamps *et al.* (19) and fluorescence measurements were converted into pH values using the calibration established in the same aforementioned study.

### Protein extract, SDS-PAGE, and *Western blotting*

To extract proteins from *L. lactis* cells, 50 ml of cell culture was centrifuged for 12 min at 1700*g* at 4 °C. The pellet was resuspended in 25 ml of ice-cold washing buffer (300 mM NaCl, 10% glycerol, 50 mM Tris-HCl pH 8.0). Cells were pelleted identically and resuspended in 1.5 ml of lysis buffer (300 mM NaCl, 10% glycerol, 1 mM PMSF, 1/2000 protease inhibitor cocktail, 2 mg/ml lysozyme, 50 mM Tris-HCl pH 8.0). Eight hundred microliters of cell suspension were mixed with 800 mg of glass beads (0.17–0.18 mm diameter), and cell lysis was performed with Precellys apparatus, 5 × 30 s at 5000 rpm. Centrifugation was performed for 12 min at 5000 rpm at 4 °C to remove cell debris, and 500 μl of supernatant was collected for further protein quantification, SDS-PAGE, and *Western blotting*.

For *Western blotting*, 20 μg of proteins were separated on SDS-PAGE gels, and *Western blotting* was carried out as previously described (19). The primary rabbit antibodies against Gdt1p were previously produced in our lab (9) and antibodies for sfpHluorin detection were purchased from Chromotek (PABG1, 1:1000 dilution). Horseradish peroxidase–coupled anti-rabbit secondary IgG antibodies and Lumi-Light Western Blotting Substrate (Roche Diagnostics) were used, and chemiluminescence was captured using an Amersham Imager 600 (GE Healthcare) with automatic exposure time for high dynamic range.

### Statistical analysis

All statistical analyses were performed using GraphPad Prism version 9.4.0. The number of replicates *N* corresponds to independent biological replicates. When possible, data sets were tested for normal Gaussian distribution with Kolmogorov–Smirnov test (α = 0.05). When *N* was too small, a Shapiro–Wilk test was used instead (α = 0.05). All data sets analyzed with these tests had a normal distribution. When a single variable was tested, a two-tailed *t* test was used to compare two data sets, and a parametric one-way ANOVA with a multiple comparison Tukey test was used for more than two data sets. If several variables were tested simultaneously, a two-way ANOVA, followed by a multiple comparison Bonferroni test, was performed. If sphericity (equal variability of differences) was not assumed, the Geisser-Greenhouse correction was applied. Except if stated otherwise, graphs are depicted with mean values, and error bars correspond to standard deviations.

## Data availability

All data are included within the manuscript and its supporting information.

## Supporting information

This article contains supporting information.

## Conflict of interest

The authors declare that they have no conflicts of interest with the contents of this article.
