# Supplementary material for: The yeast Gdt1 protein mediates the exchange of H+ for Ca2+ and Mn2+ influencing the Golgi pH
Source: J Biol Chem. 2023 Mar 22;299(5):104628. doi: 10.1016/j.jbc.2023.104628 (PMC10148156; doi:10.1016/j.jbc.2023.104628)
Supplement: Supporting information [file mmc1.pdf]

*Supporting information*

# The yeast Gdt1 protein mediates the exchange of $H^+$ for $Ca^{2+}$ and $Mn^{2+}$ influencing the Golgi pH

Antoine Deschamps, Louise Thines, Anne-Sophie Colinet, Jiri Stribny, Pierre Morsomme

UCLouvain, Louvain Institute of Biomolecular Science and Technology (LIBST), Group of Molecular Physiology, Louvain-la-Neuve, Belgium

Experiments presented in the supporting information did not require new procedures or supplemental material when compared to the main text.

**Figure S1. Expression of an intracellular pH sensor in *L. lactis* cells and *in vivo* calibration.**

(A) Excitation spectra of *L.lactis* cells not expressing the sfpHluorin (Blank), expressing the sfpHluorin alone (sfpHluorin), or together with GDT1 (*GDT1* + sfpHluorin), after induction of the proteins of interest. The emission intensity is measured at 507 nm during excitation from 350 nm to 490 nm. For all further graphs, the blank was systematically measured and subtracted before pH calculations. (B) Representative calibration experiment. Cells expressing the sfpHluorin with two hours of nisin induction were collected, washed twice and distributed in 2 ml microtubes. Cells were then centrifuged and resuspended in different pH buffers and nigericin was added at a final concentration of 0.5 µg/ml in order to permeabilize the membrane for $H^+$ and for $K^+$. Blank subtracted spectra are represented. The 390 and 470 nm excitation peaks (dashed lines) are highlighted, since they are used for ratio calculation and conversion into pH values.

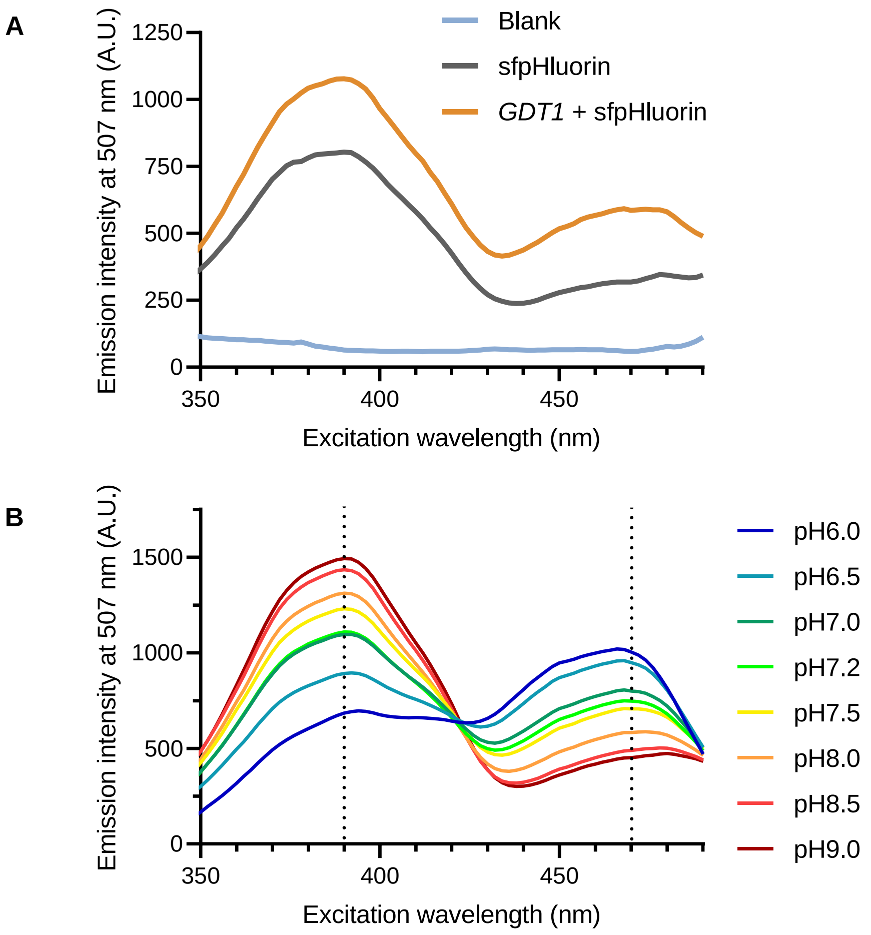

**Figure S2. Gdt1p transports protons through biological membranes.**

(A) Intracellular pH measurements over time of GDT1 expressing cells (*GDT1* + sfpHluorin) and a negative control (C- sfpHluorin) after extracellular acidification. After 60 seconds of recording the baseline, hydrochloric acid was added in the extracellular medium at a final concentration of 10 mM in order to generate a pH gradient with higher external $[H^+]$ compared to the intracellular $[H^+]$. Then, the internal pH was monitored during 4 additional minutes. $N = 5$-6. (B) Quantification of pH decrease at different time points (+ 30 sec, + 60 sec, + 120 sec, + 180 sec) compared to the pH recorded during the first 60 seconds. $N = 5$-6. The statistical analysis consists in a paired t-test, data considered are the means of each condition at the different time points.

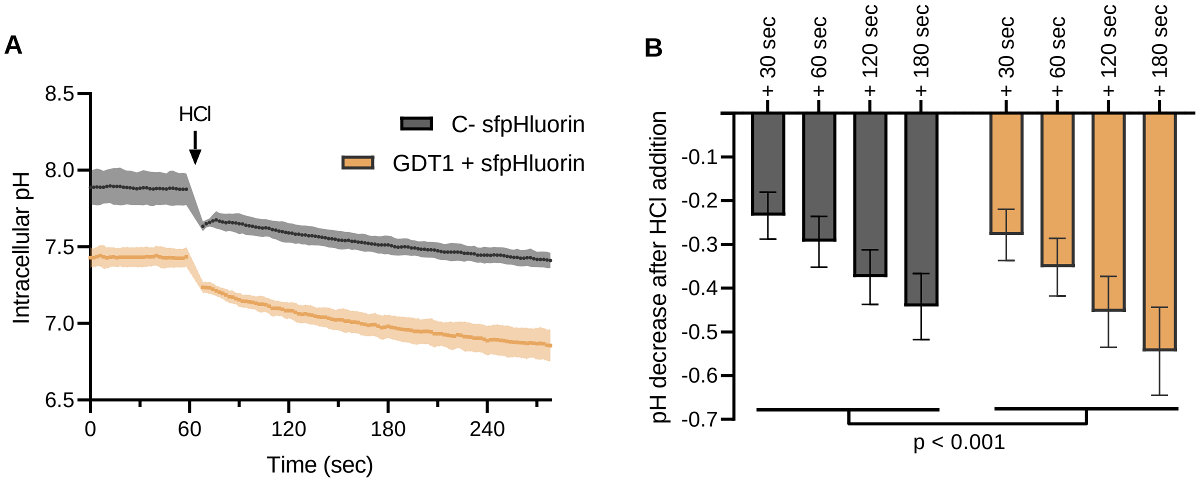

**Table S1. Complete report of p values from the statistical analysis of Figure 3.**

| **Figure 3A**<br>Addition $Ca^{2+}$ | Tukey's multiple comparisons test | Adjusted p value |
|---|---|---|
| | C- pH 6.8 vs. C- pH 7.5 | >0.99 |
| | C- pH 6.8 vs. C- pH 8.0 | 0.88 |
| | C- pH 6.8 vs. *GDT1* pH 6.8 | <0.001 |
| | C- pH 6.8 vs. *GDT1* pH 7.5 | <0.001 |
| | C- pH 6.8 vs. *GDT1* pH 8.0 | <0.001 |
| | C- pH 7.5 vs. C- pH 8.0 | 0.98 |
| | C- pH 7.5 vs. *GDT1* pH 6.8 | <0.001 |
| | C- pH 7.5 vs. *GDT1* pH 7.5 | <0.001 |
| | C- pH 7.5 vs. *GDT1* pH 8.0 | <0.001 |
| | C- pH 8.0 vs. *GDT1* pH 6.8 | <0.001 |
| | C- pH 8.0 vs. *GDT1* pH 7.5 | <0.001 |
| | C- pH 8.0 vs. *GDT1* pH 8.0 | <0.001 |
| | *GDT1* pH 6.8 vs. *GDT1* pH 7.5 | 0.70 |
| | *GDT1* pH 6.8 vs. *GDT1* pH 8.0 | <0.001 |
| | *GDT1* pH 7.5 vs. *GDT1* pH 8.0 | 0.002 |

| **Figure 3B**<br>Control $Ca^{2+}$ | Tukey's multiple comparisons test | Adjusted p value |
|---|---|---|
| | *GDT1* pH 6.8 vs. *GDT1* pH 7.5 | 0.23 |
| | *GDT1* pH 6.8 vs. *GDT1* pH 8.0 | 0.13 |
| | *GDT1* pH 7.5 vs. *GDT1* pH 8.0 | 0.90 |

| **Figure 3C**<br>Addition $Mn^{2+}$ | Tukey's multiple comparisons test | Adjusted p value |
|---|---|---|
| | C- pH 6.8 vs. C- pH 7.5 | 0.95 |
| | C- pH 6.8 vs. C- pH 8.0 | 0.68 |
| | C- pH 6.8 vs. *GDT1* pH 6.8 | <0.001 |
| | C- pH 6.8 vs. *GDT1* pH 7.5 | <0.001 |
| | C- pH 6.8 vs. *GDT1* pH 8.0 | <0.001 |
| | C- pH 7.5 vs. C- pH 8.0 | 0.99 |
| | C- pH 7.5 vs. *GDT1* pH 6.8 | <0.001 |
| | C- pH 7.5 vs. *GDT1* pH 7.5 | <0.001 |
| | C- pH 7.5 vs. *GDT1* pH 8.0 | <0.001 |
| | C- pH 8.0 vs. *GDT1* pH 6.8 | <0.001 |
| | C- pH 8.0 vs. *GDT1* pH 7.5 | <0.001 |
| | C- pH 8.0 vs. *GDT1* pH 8.0 | <0.001 |
| | *GDT1* pH 6.8 vs. *GDT1* pH 7.5 | 0.047 |
| | *GDT1* pH 6.8 vs. *GDT1* pH 8.0 | <0.001 |
| | *GDT1* pH 7.5 vs. *GDT1* pH 8.0 | 0.095 |

| **Figure 3D**<br>Control $Mn^{2+}$ | Tukey's multiple comparisons test | Adjusted p value |
|---|---|---|
| | *GDT1* pH 6.8 vs. *GDT1* pH 7.5 | 0.71 |
| | *GDT1* pH 6.8 vs. *GDT1* pH 8.0 | 0.37 |
| | *GDT1* pH 7.5 vs. *GDT1* pH 8.0 | 0.79 |
